# The sustainable development goals as a framework to combat health-sector corruption

**DOI:** 10.2471/BLT.18.209502

**Published:** 2018-06-04

**Authors:** Tim K Mackey, Taryn Vian, Jillian Kohler

**Affiliations:** a Department of Anesthesiology and Division of Infectious Diseases and Global Public Health, University of California, San Diego School of Medicine, San Diego, United States of America (USA).; b Department of Global Health, Boston University School of Public Health, Boston, USA.; c Leslie Dan School of Pharmacy, Dalla Lana School of Public Health, and Munk School of Global Affairs, University of Toronto, Ontario, Canada.

## Abstract

Corruption is diverse in its forms and embedded in health systems worldwide. Health-sector corruption directly impedes progress towards universal health coverage by inhibiting people’s access to quality health services and to safe and effective medicines, and undermining systems for financial risk protection. Corruption is also a cross-cutting theme in the United Nations’ sustainable development goals (SDGs) which aim to improve population health, promote justice and strong institutions and advance sustainable human development. To address health-sector corruption, we need to identify how it happens, collect evidence on its impact and develop frameworks to assess the potential risks and put in place protective measures. We propose that the SDGs can be leveraged to develop a new approach to anti-corruption governance in the health sector. The aim will be to address coordination across the jurisdictions of different countries and foster partnerships among stakeholders to adopt coherent policies and anti-corruption best practices at all levels. Combating corruption requires a focused and invigorated political will, better advocacy and stronger institutions. There is no single solution to the problem. Nevertheless, a commitment to controlling corruption via the SDGs will better ensure the integrity of global health and human development now and beyond 2030.

## Résumé

La corruption revêt diverses formes et mine les systèmes de santé du monde entier. La corruption dans le secteur de santé entrave directement les progrès en faveur de la couverture sanitaire universelle en empêchant l'accès de la population à des services de santé de qualité et à des médicaments sûrs et efficaces, et en ébranlant les systèmes de protection contre le risque financier. La corruption est également une thématique transversale des objectifs de développement durable (ODD) des Nations Unies, qui visent à améliorer la santé de la population, à promouvoir la justice et des institutions efficaces, et à favoriser le développement humain durable. Pour combattre la corruption dans le secteur de la santé, il est nécessaire de déterminer comment elle se produit, de collecter des données sur son impact et d'élaborer des cadres pour évaluer les risques potentiels et mettre en place des mesures de protection. Nous suggérons de tirer parti des ODD pour développer un nouveau système de gouvernance anticorruption dans le secteur de la santé. L'objectif sera d'assurer la coordination entre les systèmes juridiques des différents pays et d'encourager les partenariats entre les parties prenantes en vue de l'adoption de politiques cohérentes et de pratiques anticorruption exemplaires à tous les niveaux. La lutte contre la corruption exige une volonté politique ciblée et dynamisée, une prise de position renforcée et des institutions plus efficaces. Il n'existe pas de solution unique à ce problème. Néanmoins, un engagement en faveur de la lutte contre la corruption par le biais des ODD permettra de mieux assurer l'intégrité de la santé mondiale et du développement humain jusqu'à 2030 et au-delà.

## Resumen

La corrupción es diversa en sus formas y está incrustada en los sistemas de salud de todo el mundo. La corrupción en el sector de la salud impide directamente el progreso hacia la cobertura universal de la salud al cohibir el acceso de las personas a servicios de salud de calidad y a medicamentos seguros y eficaces, y debilitar los sistemas de protección contra los riesgos financieros. La corrupción es también un tema transversal en los objetivos de desarrollo sostenible (ODS) de las Naciones Unidas, cuyo objetivo es mejorar la salud de la población, promover la justicia y el fortalecimiento de las instituciones y promover el desarrollo humano sostenible. Para hacer frente a la corrupción en el sector de la salud, es necesario identificar cómo se produce, recopilar pruebas de su impacto y desarrollar marcos para evaluar los riesgos potenciales y establecer medidas de protección. Se propone aprovechar los ODS para desarrollar un nuevo enfoque de la gobernanza anticorrupción en el sector de la salud. El objetivo será coordinar las distintas jurisdicciones de los distintos países y fomentar las asociaciones entre las partes interesadas para adoptar políticas coherentes y prácticas óptimas de lucha contra la corrupción a todos los niveles. La lucha contra la corrupción requiere una voluntad política centrada y fortalecida, una mejor promoción y el fortalecimiento de las instituciones. No existe una única solución al problema. Sin embargo, el compromiso de controlar la corrupción a través de los ODS garantizará mejor la integridad de la salud mundial y el desarrollo humano actual y después de 2030.

## ملخص

تتنوع أشكال الفساد ويتجسد في النظم الصحية في جميع أنحاء العالم. يعيق الفساد في قطاع الصحة بشكل مباشر التقدم نحو التغطية الصحية الشاملة عن طريق إعاقة حصول الأشخاص على الخدمات الصحية ذات الجودة العالية، وكذلك حصولهم على الأدوية الآمنة والفعالة، وتقويض نظم الحماية من المخاطر المالية. ويشكل الفساد أيضاً موضوعاً شاملاً في أهداف التنمية المستدامة للأمم المتحدة (SDG)، والتي تهدف إلى تحسين صحة السكان، وتعزيز العدالة والمؤسسات القوية والنهوض بالتنمية البشرية المستدامة. ولمعالجة الفساد في القطاع الصحي، فإننا بحاجة إلى تحديد كيفية حدوثه، وجمع الأدلة حول تأثيره، ووضع أطر لتقييم المخاطر المحتملة، ووضع تدابير وقائية قيد التنفيذ. ونحن نقترح أنه يمكن الاستفادة من أهداف التنمية المستدامة لوضع نهج جديد لحوكمة مكافحة الفساد في القطاع الصحي. وسيكون الهدف هو معالجة التنسيق عبر الولايات القضائية لمختلف البلدان، وتعزيز حالات الشراكة بين الجهات المعنية لاعتماد سياسات متماسكة، فضلاً عن أفضل ممارسات مكافحة الفساد على جميع المستويات. تتطلب مكافحة الفساد إرادة سياسية مركزة ونشطة، ودعم أفضل، ومؤسسات أقوى. لا يوجد حل واحد لهذه المشكلة. ومع ذلك، فإن الالتزام بالسيطرة على الفساد من خلال أهداف التنمية المستدامة، سيضمن على نحو أفضل الاتساق بين الصحة العالمية، والتنمية البشرية الآن وما بعد عام 2030.

## 摘要

腐败的形式多种多样，并潜伏于世界各地的卫生系统内。卫生部门腐败阻碍人们获得高质量的卫生服务和安全有效的药物，破坏金融风险防范体系，直接阻碍全面卫生覆盖进程。在联合国的可持续发展目标 (SDG) 中，腐败也是一个跨领域主题，该主题旨在改善人口健康，促进公平和强有力的机构，推动人类可持续发展。为解决健康领域的腐败问题，我们需要确定其是如何发生的，收集有关其影响的证据，制定框架来评估其潜在风险并采取相关保护措施。我们建议，可以利用可持续发展目标为卫生部门的反腐败治理制定一种新的方法。其目的将是解决不同国家之间的协调问题，并促进利益攸关方之间的伙伴关系，以便在各级采取一致的政策和反腐败最佳做法。打击腐败需要集中精力和有活力的政治意愿、更好的宣传和更强有力的机构。这个问题没有单一解决方案。尽管如此，通过可持续发展目标来控制腐败的承诺，将更好地确保现在和 2030 年以后全球卫生和人类发展的完整性。

## Резюме

Коррупция имеет различные формы и внедрена в системы здравоохранения во всем мире. Коррупция в секторе здравоохранения напрямую препятствует прогрессу в обеспечении всеобщего охвата медико-санитарным обслуживанием, препятствуя доступу людей к качественным медицинским услугам и безопасным и эффективным лекарственным средствам, а также подрывает системы защиты от финансовых рисков. Коррупция также является сквозной темой в целях в области устойчивого развития (ЦУР) Организации Объединенных Наций, которые направлены на улучшение здоровья населения, содействие правосудию, укрепление институтов и обеспечение устойчивого развития человеческого потенциала. Для борьбы с коррупцией в секторе здравоохранения нам необходимо определить, как это происходит, собрать фактические данные о ее влиянии и разработать основы для оценки потенциальных рисков и принятия защитных мер. Мы предлагаем использовать ЦУР для разработки нового подхода к борьбе с коррупцией в секторе здравоохранения. Цель будет заключаться в обеспечении координации между юрисдикциями разных стран и укреплении партнерских отношений между заинтересованными сторонами для принятия согласованной политики и лучшей практики борьбы с коррупцией на всех уровнях. Борьба с коррупцией требует целенаправленной и активной политической воли, более эффективной пропаганды и укрепления институтов. Единого решения проблемы не существует. Тем не менее приверженность борьбе с коррупцией с помощью ЦУР будет лучше обеспечивать целостность глобального здравоохранения и развития человеческого потенциала в настоящее время и после 2030 года.

## Introduction

Corruption is the abuse of entrusted power for private gain.^1^ The concept of corruption in relation to global health has been defined as: “misappropriation of authority, resources, trust or power for private or institutional gain that has adverse effects on regional, local or international health systems and/or that negatively impacts individual patient and/or population health outcomes.”^2^ A study in 2013 by Transparency International, a global civil society organization working against corruption, found that in 42 out of 109 countries surveyed, more than 50% of citizens believed that the health sector in their country was corrupt or very corrupt.^3^

The exact financial cost of corruption affecting the health sector is unknown because corruption, by its nature, is often hidden.^4^ However, the scope and impact of corruption is widespread, with estimates that, on a worldwide basis, there are up to United States dollars (US$) 2 trillion in bribes paid every year in countries of all income levels.^5^ The World Health Organization (WHO) estimated that of the US$ 5.7 trillion spent on health worldwide in 2008, US$ 415 billion (7.3%) was lost to health-care fraud and abuse.^6^ Using data collected from 33 organizations in 7 countries, one study estimated global average losses from health-care fraud and abuse in 2013 to be 6.19% (US$ 455 billion of the US$ 7.35 trillion global health-care expenditure).^7^

The adverse effects of corruption are not only financial: there are societal and human costs too, especially in low-income settings. A study in 20 African countries found that a higher perceived level of national corruption was associated with poorer health, with a more detrimental impact among people of lower socioeconomic status.^8^ A study demonstrated significant association between child mortality and national perceived levels of corruption, with estimates that up to 140 000 annual child deaths could be indirectly attributed to corruption.^9^ The immediate and delayed effects of corruption on health outcomes, including higher morbidity and mortality, are due to the barriers it creates to access to health-care services, particularly for the most vulnerable groups of the population. Corruption has a negative impact on health-system quality, while distorting the allocation of countries’ health investments.^9^

Corruption also slows progress towards achieving universal health coverage (UHC), a unifying strategy to achieve the United Nations’ (UN) sustainable development goal (SDG) 3, as set out in *Transforming our world: the 2030 agenda for sustainable development.*^10^ SDG 3 focuses on ensuring healthy lives and promoting well-being for all. Specifically, corruption negatively impacts SDG 3 by impeding people’s access to quality health services and to safe and effective medicines, while also undermining systems for financial risk protection. Addressing health corruption complements the right to health, a principle enshrined in international law through the Universal Declaration of Human Rights and WHO Constitution,^11^^,^^12^ and which underpins UHC and the SDG health-related targets and indicators.

This article aims to identify and characterize the main types of health-sector corruption and explore frameworks for assessing risks of corruption and identifying protective factors. We also outline international efforts to combat health-sector corruption. Finally, we propose a new health corruption governance framework embedded within the 2030 agenda. We hope that such a framework could help catalyse international action to combat corruption in the health-care setting.

## Challenges

There are many reasons why the health sector is vulnerable to corruption.^13^^,^^14^ Important factors include the complexities of national health-care systems, which often combine both public and private health-care providers, and the large numbers and categories of people involved (patients, providers, insurers, administrators, distributors, dispensers and policymakers). Another factor is the globalized nature of the supply chain for health-care products, which increases the number of points in the system that are susceptible to corruption. The large amounts of public and private spending involved (including the high costs of administration and increased international development assistance for health programmes) also create opportunities for corruption. Finally, there are information asymmetries between actors when one party has more or better information than another, which can impact negatively health-care decision-making. These vulnerabilities that can lead to corruption can weaken health systems, waste resources and reduce the resilience of countries to health emergencies, leading to compromised coverage and access to essential health-care services.^2^^,^^14^^–^^16^

### Types of corruption

Global health-sector corruption is multifaceted and involves the jurisdictions of different countries. The United Nations Convention Against Corruption (UNCAC) is an international, legally binding treaty aimed at preventing, criminalizing and establishing international cooperation and information-sharing to fight corruption. Signatory countries to the UNCAC agree to criminalize bribery, embezzlement, misappropriation, diversion of property by public officials, trading in influence, abuse of functions and illicit enrichment as specific categories of corruption.^2^^,^^17^ Notably, bribery and embezzlement in the private sector is also criminalized, as well as money-laundering. Another form of corruption that particularly affects health-care reimbursement systems is fraud, defined as intentional deception or misrepresentation made with the knowledge that the deception could result in unauthorized benefits.^18^ The scale of corruption in the health sector can range from bureaucratic corruption (involving interactions between citizens and frontline workers or government agents) to corruption at the highest levels of policy or legislative decision-making.^19^ A WHO report^6^ identified practices ranging from theft and diversion of medicines and medical devices across the international health-supply chain, to widespread medical billing and insurance fraud for services never rendered.

Specific to the health sector, forms of corruption include: informal payments by patients to providers; absenteeism (workers who are legitimately on a payroll but are chronically absent without approval); ghost workers (non-existent individuals receiving salaries through the payroll system); reimbursement fraud (requesting insurance payments for services not rendered); dual practice (clinicians with salaries in the public sector who also maintain a private practice to divert patients or resources for their own financial gain); and improper marketing (promoting a drug for a clinical indication that is not approved for use; misleading marketing claims).^2^^,^^14^^–^^16^^,^^19^^–^^21^ These problems, however, may originate from other causes, such as unintentional errors or omissions, or may constitute only an ethics violation, not an actual crime, based on a country’s applicable laws and regulations. Other forms of health corruption are multi-jurisdictional and may involve transnational criminal networks, such as the international trade in falsified and substandard medicines, or organized criminal networks that are directly involved in health fraud schemes in multiple countries.^22^^,^^23^ Importantly, types of health corruption can occur across multiple dimensions of the health sector, with Transparency International identifying eight key areas of susceptibility: (i) health-systems governance; (ii) health-systems regulation; (iii) research and development; (iv) marketing; (v) procurement; (vi) product distribution and storage; (vii) financial and workforce management; and (viii) delivery of health-care services (Table 1).

**Table 1:** Categories of health corruption and their characteristics

Health corruption category	Description	Potential health system actors	Specific examples
Health-system governance	Corruption that undermines the governance process of policy and legislation setting in the health system for private gain	Lobbying firms Manufacturers Trade associations Insurance providers Politicians and law-makers	Lobbying activities aimed at influencing government health-care decisions and policy without integrity or transparency Undue influence over the political process to impact health policy, regulation or law Conflicts of interests associated with health-care suppliers or service providers
Health-system regulation	Corruption that undermines regulatory processes aimed at ensuring patient safety and appropriate use of health products	Regulators Manufacturers Trade associations	Inappropriate regulatory approval of health products Inappropriate inspection, accreditation, certification and product selection of health services, facilities and products Regulatory capture (when entire sections of health-care regulation are captured by select groups)
Research and development	Corruption and fraud in research and development activities for biomedical innovation	Researchers Academic institutions Manufacturers Clinical research organizations	Fraudulent research and clinical trial data Conflicts of interests between researchers and companies or sponsors Ghost-writing (when an author receives assistance with a scientific article and it is not acknowledged) Unethical practices in biomedical research Misleading research and clinical trial findings that are then disseminated or used to impact health-care services
Marketing	Corruption and fraud in marketing practices to increase profits or unduly influence prescribing or purchasing	Manufacturers Medical communication companies Health-care providers Patient and professional organizations	Gifts and other financial inducements to health-care providers False and misleading marketing claims Off-label promotion (where illegal), by marketing a drug for an indication which has not been approved Kickbacks (payments to induce or reward patient referrals or the generation of business involving health-care) Improper continuing medical education funding that involves conflict-of-interest in content or acts as a form of improper inducement
Procurement	Corruption and collusion in procurement of health products, supplies, equipment and related services	Manufacturers Distributors and wholesalers Procurement officials	Bid-rigging (when parties agree in advance to which company will win a bid) Collusion between bidders for contracts Influencing drug formulary decisions Unfulfilled delivery of contracts
Product distribution and storage	Corruption as it relates to the distribution, transport, and storage of medicines and other health commodities	Distributors and wholesalers Pharmacies and other dispensers Regulators Unauthorized manufacturers	Theft and diversion of products Expiration, spoilage and adulteration of products Falsified and substandard medicines Corruption that leads to medicine stock-outs
Financial and workforce management	Corruption that impacts financing and workforce management and that limits health-care services	Health administrators Health-care providers Donors Politicians and public servants	Theft, embezzlement and misallocation of health-care funds Unjustified absenteeism (workers who are legitimately on a payroll, but are chronically absent without approval) Dual practice (when clinicians who have salaries in the public sector also maintain a private practice to divert patients or resources for their own financial gain) Improper billing or payments, upcoding (when a provider bills for a service that is more expensive than the one performed), and false claims Self-referral (when a health-care provider refers a patient to an entity they have a financial relationship with) Ghost workers (non-existent individuals receiving salaries through the payroll system) Inappropriate selection for jobs, promotions and training
Delivery of health-care services	Any type of corruption that directly impacts the quality and level of care offered to patients from health-care providers	Health-care providers Health-care administrators Patients	Medically unnecessary referrals and treatment Informal payments to health-care providers Dual practice Medical malpractice Favouritism or nepotism (favouring someone in health-care decision-making or resource allocation) Overcharging for services Manipulation of data (deliberate falsification or manipulation of data concerning biomedical research or clinical trials)

Source: Petkov & Cohen.^24^

### Existing data on corruption

Recent reviews have synthesized evidence of corruption in the health sector in different countries and contexts.^14^^,^^20^^,^^25^ Corruption is often measured using surveys of peoples’ perceptions or experiences (e.g. Transparency International’s global corruption barometer survey)^3^ or social audits (e.g. household surveys that include questions about perceptions and experiences with corruption). There have also been special studies focused on problems such as ghost workers or unjustified absenteeism.^4^^,^^14^^,^^16^^,^^26^^,^^27^ Other official sources are investigative reports by oversight agencies, such as a national agency responsible for auditing government revenue and spending or an ombudsman’s office comprised of officials charged with investigating complaints of corruption. Reports generated by the media and investigative journalism also can serve as important alerts and uncover corruption for further investigation. Other data sources, including health commodity procurement prices and enforcement data (e.g. number and amount of settlements for corruption-related prosecutions), act as useful proxy indicators.

Additionally, qualitative studies documenting people’s lived experience with corruption in the health sector provide specific details that go beyond perception surveys. For example, a systematic review of 38 research studies found a huge range in the proportions of patients who reported making informal payments in the health-care system (from 2% to 80%).^28^ Similarly, surveys in Africa found that the percentage of people paying bribes to obtain health-care services ranged from 1% of 1200 people surveyed in Botswana to 69% of 1199 people in Liberia.^29^ In a study of the 28 Member States of the European Union, 8704 (31%) of 28 080 respondents thought that corruption was widespread in their health-care system. Yet only 872 (4%) of 21 789 respondents reported having to give an extra payment, valuable gift or make a donation to the hospital to access care. The highest proportions of informal payments were in Greece (13%), Hungary (17%) and Romania (19%; numbers not reported).^30^

Overall, variability in data quality, sources and methods of analysis and a general lack of systematic and standardized data collection make it hard to generalize the actual prevalence and overall trends in different categories of health corruption across different countries.

### Frameworks for assessing corruption

Several frameworks have been proposed for assessing the potential risks and protective factors for health-sector corruption. Among these are Transparency International’s *Global corruption report 2006*^4^ and the Organisation for Economic Co-operation and Development’s (OECD) 2017 report^20^ on intentional integrity violations (a more inclusive term than corruption). Transparency International’s framework considers the relationships among five major categories of people: (i) government regulators (ministries, parliaments, commissions); (ii) payers (social security, government, private insurers); (iii) providers (hospitals, doctors, pharmacists); consumers (patients); (iv) drug and equipment suppliers (pharmaceutical and medical device companies); (v) and other suppliers (hospital construction companies). This framework illustrated how fragmentation and delegation of responsibilities through a decentralized health system can increase the system’s vulnerability to corruption.

In 2017 a European Commission study was made to establish the types of health corruption that may put health systems at heightened risk.^25^ Three issues were identified: (i) privileged access to medical services (including informal payments and misuse of privileged information); (ii) improper marketing (including for market authorization and reimbursement); and (iii) dual practice.

A 2008 review of health-sector corruption presented a model of factors that occur at the individual level, driven by pressures, opportunities and rationalizations to abuse. These individual-level drivers then translate to risks for corruption that manifest at the larger health-systems level and are driven by other macro risk factors such as weak citizen voices (limited participation by citizens in planning and monitoring government services) and monopoly (limiting citizen choices and forcing interaction with corrupt agents).^16^ This framework conceptualized corruption as the result of individual actions and systems-level problem, requiring strategies that recognize and address risk factors at both these levels.^16^^,^^31^

Anti-corruption strategies and tactics to control corruption in the health sector often focus on protective factors, including good governance approaches centred on the rule of law, transparency, accountability and participation. Transparency, in particular, is key to mitigating the risks of corruption by ensuring information is publicly available, as in e-government (i.e. use of electronic devices to provide services to citizens), open contracting and e-procurement approaches.^32^^–^^34^ Accountability to the public by public officials is also essential to foster trust in public institutions, the decision-making processes and governance, with the overall aim of assessing the achievement of goals laid out by government against the standards and commitments made.^35^ Importantly, these good governance concepts have clear linkages (e.g. accountability is difficult to ascertain without transparency) and, independently, accountability or transparency alone cannot sufficiently curb corrupt practices.^36^^,^^37^

## International efforts

Current international efforts to fight health corruption are built on strategies that emerged as early as the 1970s. For example, the United States Foreign Corrupt Practices Acts was enacted due to investigations by the United States Securities and Exchange Commission and United States Senate sub-Committee on Multinational Corporations. These investigations uncovered over US$ 300 million in illegal payments by over 400 American companies, including pharmaceutical and health-care corporations.^38^ In 1999, the OECD’s Anti-Bribery Convention was adopted internationally to criminalize bribery of foreign public officials in international business transactions. The Convention followed ministerial decisions to make anti-corruption a policy priority for the World Bank and the International Monetary Fund. As a first anti-corruption step by the UN, the non-binding UN Global Compact at the end of the 1990s established principle 10 to encourage businesses to fight corruption. This was followed by other anti-corruption activities from the International Chamber of Commerce, which rewrote its *Rules on combating corruption* in 2011,^39^ and the World Economic Forum, whose Partnering Against Corruption initiative was launched in 2004.^40^

It was not until 2005, however, that the UN’s global anti-corruption efforts came to the fore, with the adoption of the UNCAC by the United Nations Office of Drugs and Crime (UNODC). The UNCAC created the first global treaty aimed at preventing, criminalizing, controlling and strengthening international cooperation against corruption in all its forms.^2^ Near universal adoption of the UNCAC by UN Member States also helped support anti-corruption programmes in many multilateral development institutions, such as the World Bank, regional development banks (as well as leading bilateral aid agencies) and the United Nations Development Programme (UNDP). These institutions identified corruption as one of the biggest obstacles to social and economic development and adopted good governance and anti-corruption programmes in response.

Specific to health, the WHO launched the Good Governance for Medicines programme in 2004, which sought to prevent corruption by promoting transparency in the pharmaceutical sector.^41^ More recent developments from the WHO include an update to its Good Governance for Medicines programme tool and a workstream led by the health-systems governance and financing team and the gender, equity and rights team. This project aims to advance work on strengthening transparency and accountability in health systems, including specifically linking anti-corruption efforts to UHC.

Global health partnerships, such as the Global Fund to Fight AIDS, Tuberculosis and Malaria, have also attempted to address corruption more proactively. This followed from damaging reports of misallocation, fraud, collusion in bidding and drug theft in their country programmes.^13^^,^^42^^,^^43^ Global Fund anti-corruption initiatives include the I Speak Out Now campaign and its Two to One penalty that withholds double the amount of future grant funding when lost funds are not recovered. Yet, despite a variety of anti-corruption programmes and initiatives in place from different stakeholders, a recent 2016 Cochrane review found that no studies met their criteria for establishing empirical evidence of the effect of anti-corruption interventions in the health sector.^44^ The lack of evidence of effectiveness may be due in part to inadequate enforcement, particularly in the context of multi-jurisdictional corruption that requires international cooperation and coordination.

## Policy proposal

The SDGs offer a valuable opportunity and governance mechanism to tackle health corruption. Whereas the health-related millennium development goals focused primarily on infectious diseases and maternal and child health, the SDGs now explicitly declare promoting healthy lives and combating corruption as central pillars of the international policy and global governance. The SDGs can therefore be an organizing framework for international action against multi-jurisdictional health corruption. Nevertheless, the global health community needs to develop consensus around definitions and categories of corruption, standardize and enable collection of more robust data, and deploy tools to assess risk and protective factors at the individual and systems level.

To better understand the interplay between these interdependent SDG drivers of health, social justice and economic growth, we mapped the SDG goals, targets and indicators associated with health corruption (Table 2 and Table 3). We included SDG goal 3 targets 3.8 (achieving UHC including access to quality essential health-care services and medicines), 3c (increasing health-care financing and retention of health workforce) and 3d (strengthening capacity of countries to respond to global health risks), along with four specific SDG indicators on how progress towards targets are measured. SDG goal 16 includes targets 16.5 (substantially reduce bribery and corruption) and 16.6 (develop effective, accountable and transparent institutions at all levels) and three indicators. We also provide practical examples of categories of health corruption that impact each SDG target. Our most important finding is that none of the SDG goal 3 or goal 16 associated targets or indicators addresses health corruption directly.

**Table 2:** Examples of corruption affecting sustainable development goal 3 targets

SDG 3 targets^a^	SDG indicators^b^	Associated examples of health-sector corruption
3.8: Achieve universal health coverage, including…access to quality essential health-care services and access to safe, effective, quality and affordable essential medicines and vaccines for all	3.8.1: Coverage of essential health-care services 3.8.2: Number of people covered by health insurance or public health system per 1000 population	• Theft and embezzlement of health-care funds • Fraud and abuse in health-care payments and services • Corruption in procurement of health commodities and services • Corruption in product approval and facility certification • Falsified and substandard medicines • Fraudulent or misleading research • Improper inducements^c^ • False or misleading marketing • Informal payments to health-care providers • Overcharging and unnecessary referrals and services
3.c: Substantially increase health financing and the recruitment, development, training and retention of the health workforce in developing countries and small island developing States	3.c.1: Health worker density and distribution	• Unjustified absenteeism^d^ • Improper professional accreditation • Embezzlement and misuse of national and donor funds • Inappropriate selection, promotion and training of staff • Private use of public time, equipment or facilities
3.d: Strengthen the capacity of all countries, in particular developing countries, for early warning, risk reduction and management of national and global health risks	3.d.1: International Health Regulations capacity and health emergency preparedness	• Collusion in contracting^e^ • Unfulfilled contract delivery • Theft and diversion • Embezzlement of emergency funds • Ghost workers^f^ during health emergencies

SDG: sustainable development goal.

^a^ SDG 3 targets from Transforming our world: the 2030 agenda for sustainable development.^10^

^b^ Indicators from SDG target 3 that are impacted by health corruption.

^c^ Inducements include gifts and payments to health-care providers that can impact clinical care and access to services.

^d^ Absenteeism concerns workers who are legitimately on a payroll, but are chronically absent without approval.

^e^ Collusion in contracting is when there is a secret agreement between suppliers to conspire and commit actions to deceive a competitive bidding/tender process.

^f^ Ghost workers are non-existent individuals receiving salaries through the payroll system.

**Table 3:** Non-health sustainable development goals with potential application to health-sector corruption

SDG goals and targets^a^	SDG indicators	Implications for health-sector corruption
16.5: Substantially reduce corruption and bribery in all their forms	16.5.1 and 16.5.2: Proportion of persons [or businesses] who had at least one contact with a public official and who paid a bribe or were asked to bribe during the previous 12 months	Could be used to measure how many people have paid a bribe in the public health sector
16.6: Develop effective, accountable and transparent institutions at all levels	16.6.1: Primary government expenditures as a proportion of original approved budget, by sector 16.6.2: Proportion of the population satisfied with their last experience of public services	Could be used to measure misallocation of health-sector funds
17.14: Enhance policy coherence for sustainable development	17.14.1: Number of countries with mechanisms in place to enhance policy coherence of sustainable development	Need to establish policy coherence around international and regional laws, regulations, and enforcement against health-related corruption
17.16: Enhance the Global Partnership for Sustainable Development, complemented by multistakeholder partnerships that mobilize and share knowledge, expertise, technology and financial resources, to support the achievement of the SDGs in all countries, in particular developing countries	17.14.1: Number of countries reporting progress in multistakeholder development effectiveness monitoring frameworks that support the achievement of the SDGs	Need to establish multistakeholder partnerships that monitor progress towards these goals specifically in the health sector

SDG: sustainable development goal.

^a^ SDG 3 targets from *Transforming our world: the 2030 agenda for sustainable development*.^10^

In Table 4 we formulate examples of cross-cutting SDG sub-indicators that could be deployed to prevent, control and fight corruption to improve public health and we map them to existing SDG targets and indicators. These sub-indicators focus on addressing and measuring: (i) bribery and its impact on health-care access; (ii) health-care funds lost to fraud, abuse, misuse and embezzlement; (iii) corruption and its impact on quality and access to medicines (including falsified and substandard medicines); (iv) the effect of investment in health strengthening and good governance; and (v) the negative interaction between corruption and global health security. These sub-indicators also provide examples of how data could be collected, analysed and measured, using different methods and tools. Such tools include data from surveys, community monitoring, prosecutions and enforcements, audits, public health surveillance and monitoring and evaluations.

**Table 4:** Examples of shared sustainable development goal sub-indicators with potential for measuring health-sector corruption

Cross-cutting shared SDG goals and targets^a^	Shared SDG indicators	Possible tools for measuring health-sector corruption
3.8 and 16.5: (health-care access and bribery)	Proportion of persons who paid or were asked to pay a bribe or who made an informal payment] for public or private health services. Amount of US$ recovered in health-systems-related fines, penalties and settlements	Survey data Community monitoring Social media data-mining and surveillance Data on fraud and abuse prosecutions and settlements Reports and data from audits Monitoring and evaluation and programme evaluation with indicators for bribes and corruption E-government and e-procurement approaches
3.c and 16.6: (health-care workforce capacity and transparency)	Proportion of national health budget and official development assistance committed for health system strengthening, transparency initiatives, and good governance	Community monitoring Audits Data on governance expenditures and official development assistance for health
3.d and 16.5: (health emergencies and bribery)	Proportion of emergency fund expenditures with appropriate documentation	Audits Monitoring and evaluation, programme evaluation, and measuring progress towards indicators for bribes and corruption in the health-care sector Counterfactual impact evaluation designs^b^
3.8, 16.5 and 17.14: (policy coherence for health bribery and corruption)	Number of countries implementing the UNCAC provisions specific to the health sector	Monitoring implementation of the UNCAC Comparative anti-corruption policy and law analysis studies
3.8, 16.6 and 17.16: (multistakeholder partnership focused on anti-corruption in the health sector)	Amount of support and participation by countries, international organizations, and civil society in health anti-corruption partnerships	Funding commitments to SDGs related to health corruption Number of partnerships created that focus on health corruption

SDG: sustainable development goal; UNCAC: United Nations Convention Against Corruption; US$: United States dollars.

^a^ SDG 3 targets from *Transforming our world: the 2030 agenda for sustainable development*.^10^

^b^ Counterfactual impact evaluation measures impact against those not receiving a policy intervention compared to those that have been exposed to the intervention.^45^

Finally, SDG goal 17, which focuses on multistakeholder partnerships, is crucial to putting into practice a shared agenda in global health governance against corruption. Specifically, targets 17.14 (enhancing policy coherence) and 17.16 (enhancing global partnership around SDGs by complementing with multistakeholder partnerships) can help focus and advocate for needed attention on corruption risk in the health sector. This process begins with UN specialized agencies, international treaties (e.g. such as monitoring implementation of the UNCAC in the context of health) and anti-corruption programmes at different levels, aligning their policies more coherently though standardized definitions and use of best practices as part of target 17.14.

Coherence across global policy can be achieved by promoting best practices in anti-corruption, good governance, audit, transparency and accountability, and integrating them in policy instruments. These best practices include national health policies, strategies and plans; capacity-building activities, such as health-system strengthening efforts; and monitoring and evaluation, such as health sector assessments. Furthermore, given the lack of empirical data supporting anti-corruption interventions, there is an immediate need to conduct robust evaluations of the effectiveness and impact of different domestic and international anti-corruption laws (such as the Foreign Corrupt Practices Act of the United States, the Bribery Act 2010 of the United Kingdom of Great Britain and Northern Ireland and the UNCAC) that are used to control health-related corruption in all its forms.^2^^,^^13^

Finally, relevant UN institutions could mobilize this SDG-focused agenda by building global multistakeholder partnerships aimed at achieving shared goals of SDGs 3 and 16 as part of target 17.16. Initial participating organizations could include UNDP, WHO, UNODC (which promotes the UNCAC and houses UNCAC’s Conference of the State Parties) and the World Bank. Additionally, non-state civil society organizations such as Transparency International and the U4 Anti-Corruption Resource Centre^15^ should also have some form of participation in SDG partnerships to provide insights on community engagement, conduct anti-corruption research and act as a resource for anti-corruption training.

## Conclusion

Corruption in the health sector is a major challenge to advancing population health, social justice, shared prosperity and sustainable development, all of which are core pillars of the SDGs. Efforts to prevent corruption need to begin with international consensus, recognizing the unique and destructive consequences of health-sector corruption. We offer a blueprint for how global stakeholders can use the SDG framework to advocate and prioritize combating corruption’s impact on health. We call for the UN’s Inter-agency Expert Working Group on SDG Indicators to partner with the WHO to hold consultations on the formation of a multistakeholder health-corruption partnership under the SDGs.^13^ We argue that combating corruption should be a core value of the SDGs, due to its links to human rights, equity, economic development and UHC. Combating the disease of corruption is critical to ensuring the sustainability of global health and human development in 2030 and beyond.
